# The community background alters the evolution of thermal performance

**DOI:** 10.1093/evlett/qrae007

**Published:** 2024-03-16

**Authors:** Joseph Westley, Francisca C García, Ruth Warfield, Gabriel Yvon-Durocher

**Affiliations:** Environment and Sustainability Institute, The Centre for Ecology and Conservation, University of Exeter, Penryn, Cornwall, United Kingdom; Environment and Sustainability Institute, The Centre for Ecology and Conservation, University of Exeter, Penryn, Cornwall, United Kingdom; Red Sea Research Center (RSRC), Division of Biological and Environmental Science and Engineering (BESE), King Abdullah University of Science and Technology (KAUST), Thuwal, Saudi Arabia; Environment and Sustainability Institute, The Centre for Ecology and Conservation, University of Exeter, Penryn, Cornwall, United Kingdom; Environment and Sustainability Institute, The Centre for Ecology and Conservation, University of Exeter, Penryn, Cornwall, United Kingdom

**Keywords:** thermal adaptation, microbial communities, global warming, thermal performance, interspecific interactions

## Abstract

Microbes are key drivers of global biogeochemical cycles, and their functional roles arey dependent on temperature. Large population sizes and rapid turnover rates mean that the predominant response of microbes to environmental warming is likely to be evolutionary, yet our understanding of evolutionary responses to temperature change in microbial systems is rudimentary. Natural microbial communities are diverse assemblages of interacting taxa. However, most studies investigating the evolutionary response of bacteria to temperature change are focused on monocultures. Here, we utilize high-throughput experimental evolution of bacteria in both monoculture and community contexts along a thermal gradient to determine how interspecific interactions influence the thermal adaptation of community members. We found that community-evolved isolates tended toward higher maximum growth rates across the temperature gradient compared to their monoculture-evolved counterparts. We also saw little evidence of systematic evolutionary change in the shapes of bacterial thermal tolerance curves along the thermal gradient. However, the effect of community background and selection temperature on the evolution of thermal tolerance curves was variable and highly taxon-specific,with some taxa exhibiting pronounced changes in thermal tolerance while others were less impacted. We also found that temperature acted as a strong environmental filter, resulting in the local extinction of taxa along the thermal gradient, implying that temperature-driven ecological change was a key factor shaping the community background upon which evolutionary selection can operate. These findings offer novel insight into how community background impacts thermal adaptation.

## Teaser

Previous research investigating how microbes evolve in response to changes in temperature has focused on single-taxon cultures (monocultures). Here, we evolve five bacteria, either combined in communities or separately in monocultures, along a thermal gradient. We show for the first time that the presence of other taxa alters how the bacteria evolve in response to changes in temperature. This research can help us to understand how microbial ecosystems may adapt to a warming climate.

## Introduction

Microbial communities make key contributions to the functioning of ecosystems (Falkowski et al., 2008) and play critical roles in global elemental cycles (Davidson & Janssens, 2006; Giorgio & Duarte, 2002; Goericke & Welschmeyer, 1993; Philippot et al., 2013; Williams, 1981). Temperature exerts strong control over subcellular metabolic processes, which in turn determine the contribution of microbial communities to ecosystem functioning and biogeochemical cycling (Duffy et al., 2021; Mahecha et al., 2010; Qi et al., 2002; Yvon-Durocher et al., 2010, 2012, 2014). Therefore, there is an urgent need to understand the mechanisms that shape how microbial communities will respond to rising global temperatures.

Over short time scales, microorganisms exhibit acute responses to changes in temperature (Antoniou et al., 1990; Bakermans & Nealson, 2004; Margesin, 2009; Pietikäinen et al., 2005). However, given their short generation time, natural microbial communities are predicted to respond to long-term global warming via evolutionary adaptation of individual community members (O’Donnell et al., 2018; Padfield et al., 2016; Tian et al., 2022), ecological sorting of the standing community diversity (Garcia et al., 2022), and invasion by taxa that are better preadapted to the novel thermal environment (Vezzulli et al., 2012, 2013).

In monoculture thermal adaptation experiments, it has been shown that selection favors a shift in the thermal dependence of key metabolic traits, such as downregulation of respiration and photosynthesis in high-temperature evolved phytoplankton (Padfield et al., 2016), a higher thermal optimum in high temperature evolved diatoms (O’Donnell et al., 2018), narrower thermal performance curves (TPCs) in *Escherichia coli* evolved at a constant temperature (Cooper et al., 2001), a higher thermal minimum in high temperature evolved bacteria (Tian et al., 2022), and improved tolerance of temperature fluctuations in bacteria evolved in a fluctuating thermal environment (Saarinen et al., 2018). However, wild microbes inhabit highly speciose communities (Curtis et al., 2002), and owing to a notable lack of studies that utilize experimental evolution of thermal tolerance in a community context, it is unclear how the community background might impact the evolutionary trajectories of microbial thermal adaptation.

Several studies have provided evidence that interspecific competition in microbial communities can impact environmental adaptation. Theoretical models have predicted that interspecific competition can constrain adaptive evolution in the face of environmental change via reduced population sizes and reduced selective pressure on tracking the optimum resource niche (Johansson, 2008). Experimental studies have shown that community members have reduced rates of adaptation to novel CO_2_ concentrations (Collins, 2011), low pH (Scheuerl et al., 2020), new food sources (Lawrence et al., 2012), and even typical laboratory conditions (Castledine et al., 2020), compared with their monoculture evolved counterparts. The existence of trade-offs between adaptation to abiotic conditions and community background is an intuitive and frequently hypothesized explanation for these findings. If such trade-offs exist between adaptation to the thermal environment and heterospecific imposed selection pressures, a microbial community member may experience more constrained adaptation to temperature than they would in a monoculture.

Diverse communities can impact their members’ evolutionary response to increased temperature via mechanisms beyond competition-imposed selective trade-offs. Heterospecifics can be sources of beneficial mobile genetic elements. For example, the transmissible locus of stress tolerance (tLST) is a highly conserved and widely transmitted genomic island that codes for a number of gene products, including small heat shock proteins (Kamal et al., 2021). Natural microbial communities can also contain nonprokaryotic organisms, such as bacteriophages (phages) that parasitize bacteria and archaea. As it has been shown that differences in the TPCs of phages and their hosts lead to acute shifts in the latter’s TPC (Padfield et al., 2020), phage presence could also modulate thermal adaptation over longer timescales. Protist bacterivores are also widespread and functionally important members of microbial communities, and recent research has shown that the presence of predators interacts with temperature to regulate microbial community respiration (Rocca et al., 2022).

Despite the urgent need to understand how microbial ecosystems will adapt in response to warming, there remains considerable ambiguity surrounding how community context influences the evolution of thermal performance. Here, we address this gap by evolving five taxa across a range of temperatures in both monocultures and communities. Using a high-throughput growth assay, we quantified the TPCs for each evolved lineage, allowing us to determine how the community background shapes the evolution of thermal performance.

## Results and discussion

### All taxa analysis

To investigate the influence of the community background on the evolution of thermal performance, we designed an experiment whereby five bacterial taxa were evolved in two treatment groups, “monoculture” and “community,” for ~100 generations. In the monoculture treatment group, each taxon was evolved without the presence of heterospecifics at eight different “evolution temperatures” (ranging from 15 °C to 42 °C) for ~100 generations. In the community treatment group, all five taxa were combined into communities and evolved at the same eight evolution temperatures as the monoculture treatment group. At the end of the experiment, each evolved lineage from every experimental unit was isolated, and their TPC was quantified by assaying the maximum growth rate across the thermal gradient (i.e., taxon A, evolved at 15 °C in the monoculture treatment group, would be grown at all eight temperatures ranging from 15 °C to 42 °C).

We used a generalized additive mixed-effects model (GAMM) approach to analyze the TPCs of the isolates. In this experiment, our principal aim was to quantify whether evolving in a community context or in monoculture affects the evolution of thermal performance. Our target with the analysis of the TPCs was, therefore, to determine whether the shape—i.e., the nature of the relationship between growth rate and temperature—and the elevation—i.e., the average growth rate across the TPC—differed between monoculture and community evolved isolates. A GAMM allowed us to achieve these aims within a highly flexible statistical modeling framework. First, the GAMM captures the nonlinear shape of the TPCs using a smoothing function (Venables & Ripley, 2002). Importantly, unlike mechanistic models of TPCs (Schoolfield et al., 1981), the GAMM makes no assumptions about the shape of TPCs and, therefore, affords additional flexibility to capture differences among the taxa and treatment groups. Second, the GAMM facilitates modeling the hierarchical structure of the data—i.e., taxon-level TPCs nested with the broader aggregate response at the treatment group level.

**Figure 1.**
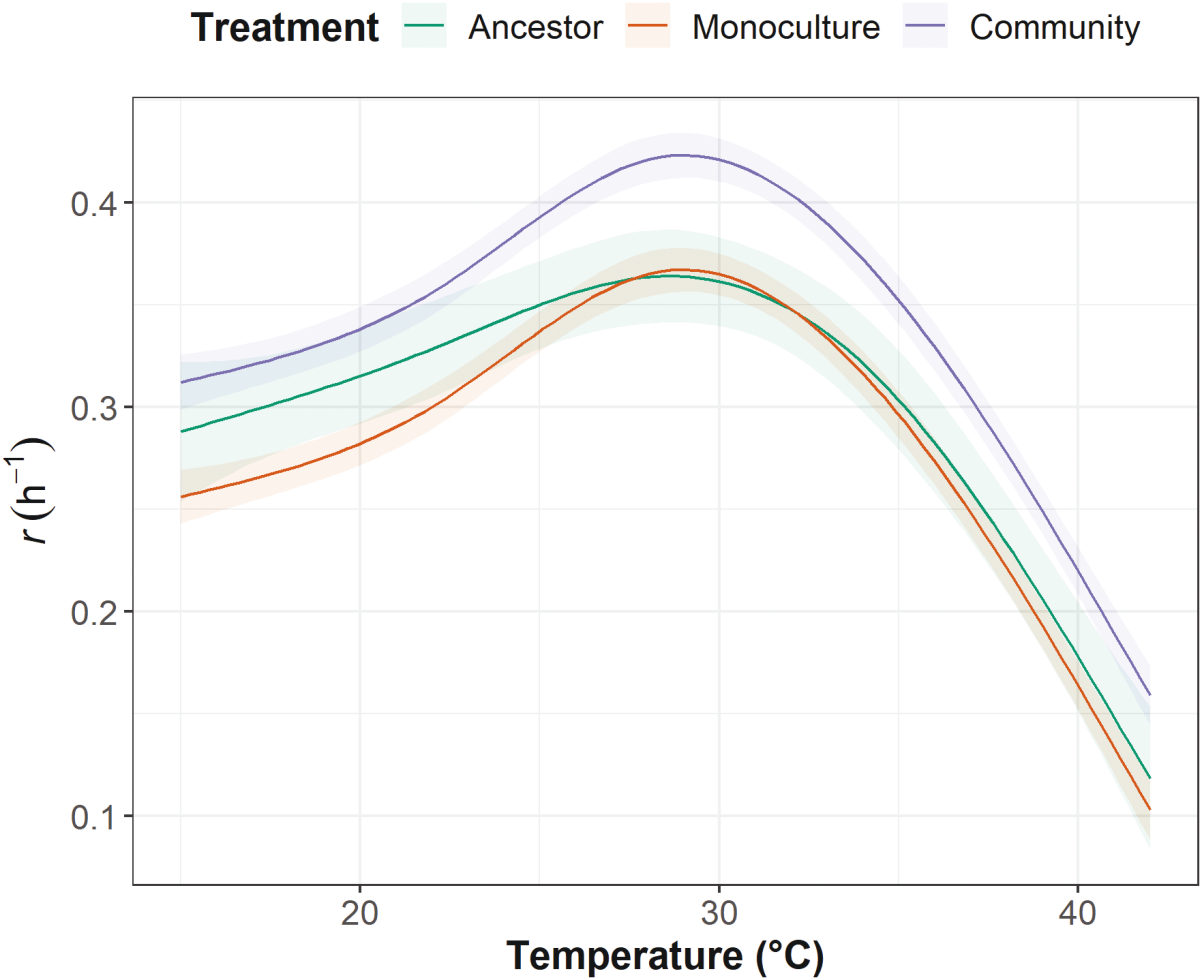
The relationship between maximum growth rate [*r*(h^−1^)] and growth temperature (°C), including all five taxa evolved in monocultures (orange), communities (purple), and the ancestors to the evolved isolates (green). For evolved isolates, all evolution temperatures are combined. Lines denote the model-fitted values, while ribbons denote the standard errors. Evolved isolate and ancestor curves are produced from separate models.

We found that there was a significant difference in the intercepts of the TPCs between isolates that evolved in a community context and isolates that evolved in monoculture (Figure 1; Table 1). Bacteria isolated from community settings had higher maximum growth rates across the TPC (estimated marginal means [centered on mean of growth temperature] of growth rate for community evolved is 0.421 ± SE 0.011, and for monoculture evolved is 0.365 ± SE 0.011). Allowing the smoothing term on temperature to vary between monoculture and community-evolved isolates did not significantly improve the fit of the model (Table 1), implying that there was no difference in the shape of the TPC between these groups. There was also no significant effect of evolution temperature or significant interaction between community context and evolution temperature on the intercept (see Table 1 “All taxa” for ΔAICc values for removal of each fixed effect and Table 2 for Tukey’s adjusted *p*-values and effect sizes).

**Table 1.** The significance of fixed effects on maximum growth rate [*r*(h^−1^)] for the five taxa modeled separately as well as the model containing all taxa combined.

	*Pseudomonas* spp.	*Serratia* spp.	*Aeromonas* spp.	*Herbaspirillum* spp.	*Janthinobacterium* spp.	All taxa
Treatment intercept	20.0739	22.0284	41.5687	7.696	NS	14.501
Evotemp intercept	12.5241	8.2962	NS	7.62	NS	NS
Smooth varies by treatment	NS	14.7586	9.9028	10.954	33.646	NS
Treatment: Evotemp	NS	NS	NS	8.728	NS	NS

All models are generalized additive mixed-effects models (GAMMs). Values are ΔAICc for the removal of the respective fixed effect from the lowest AICc model. “NS” is “nonsignificant” and denotes that the respective fixed effect was not present in the best model for that taxon. “Evotemp” refers to the evolution temperature an isolate was evolved at, “Treatment” denotes whether an isolate is community evolved, monoculture evolved, or ancestral (for the “All taxa” analysis, Treatment refers to only community evolved, and monoculture evolved). “intercept” denotes an intercept effect while “Smooth vary by Treatment,” denotes whether the smoothing term was allowed to vary between treatments groups.

**Table 2.** Tukey’s adjusted *p*-values (*p*) and pairwise differences (effect size) for pairwise comparisons of all treatment-level intercepts.

	*Pseudomonas* spp.		*Serratia* spp.		*Aeromonas* spp.		*Herbaspirillum* spp.		*Janthinobacterium* spp.		All taxa	
	*p*	Effect size	*p*	Effect size	*P*	Effect size	*p*	Effect size	*p*	Effect size	*p*	Effect size
Ancestor—Monoculture	**<0.001**	−0.0678	**0.019**	−0.0907	0.911	0.0222	**<0.001**	−0.0822	NA	NA	NA	NA
Ancestor—Community	**<0.001**	−0.1436	**<0.001**	−0.1262	**<0.001**	−0.2308	**<0.001**	−0.0783	NA	NA	NA	NA
Monoculture—Community	**<0.001**	−0.0758	0.379	−0.0355	**<0.001**	−0.253	0.931	0.00387	NA	NA	**<0.001**	−0.056

Bold values denote statistically significant pairwise differences.

We additionally modeled the relationship between temperature and growth rate for the ancestors, allowing us to make comparisons with the evolved isolates. We found that the ancestor had a similar estimated marginal mean growth rate to the monoculture-evolved isolates (0.364 ± SE 0.022). This suggests that the growth rate of the monoculture-evolved isolates changed relatively little compared to the ancestor, while by contrast, the community-evolved isolates evolved a higher growth rate (see Statistical analysis section for why the ancestor was not included as an additional level of treatment group).

We hypothesize that higher growth rates in the community-evolved treatment group are driven by interspecific competition, which selects for more rapid resource acquisition and, thus, a higher maximum growth rate. Although a key prediction of ecological theory is that the strength of intraspecific competition will exceed that of interspecific competition (Adler et al., 2018; Goldberg & Barton, 1992; Macarthur & Levins, 1967), the community-evolved isolates will have been subjected to both intra- and interspecific competition, while monoculture evolved isolates face only intraspecific competition. The additional effect of interspecific competition could have driven the evolution of a higher maximum growth rate in the community setting compared to that in monoculture. This result is consistent with previous research demonstrating the competitive advantage provided by a higher growth rate (Ketola et al., 2016; Winkler et al., 2017). However, the evolution of a higher growth rate in a community context is not ubiquitous, with another study finding the opposite effect; community-evolved isolates had reduced maximum growth rates due to cross-feeding specialization (when one taxon feeds on waste products of another taxon) occurring in the community evolved isolates, a strategy that could not be utilized when growth assayed in monoculture (Lawrence et al., 2012). It is important to note that although we observe higher growth rates in the community evolved isolates when they are assayed in monoculture, it is possible that their actual growth rates in community settings would be reduced by antagonistic interactions with other taxa, as observed in O’Donnell et al. (2018).

### Within-taxon analyses

**Figure 2.**
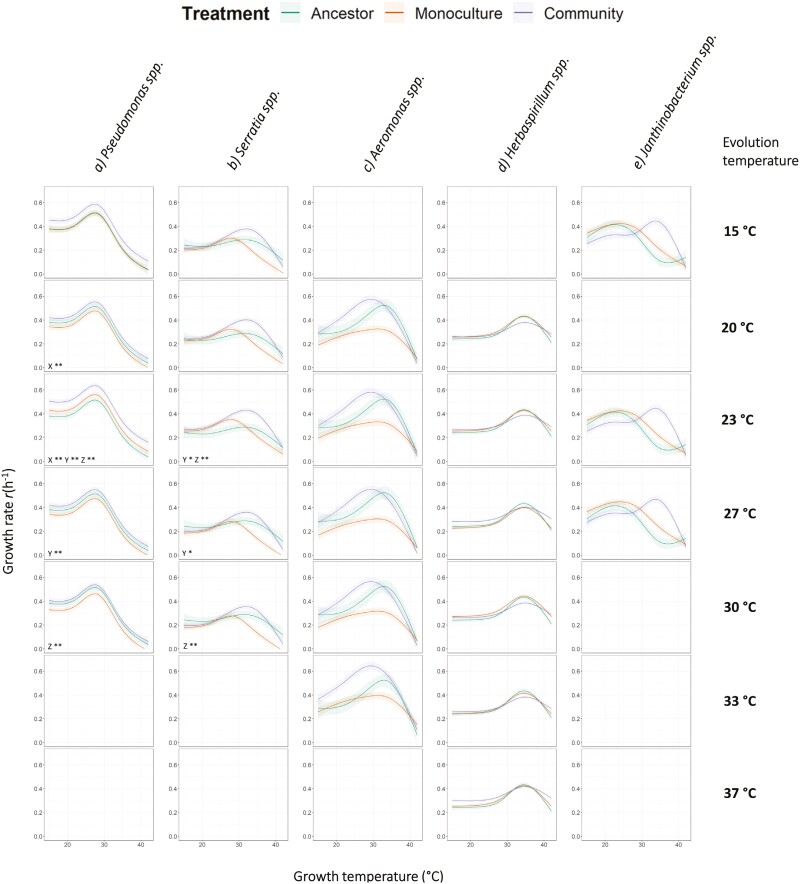
The relationships between maximum growth rate [*r*(h^−1^)] and growth temperature (°C) for isolates evolved in monocultures (orange), communities (purple), and the ancestors to the evolved isolates (green). Lines denote the model-fitted values, while ribbons denote the standard errors. Each row of plots has different evolution temperatures, and each column (A–E) is a different taxon. Empty plots denote that the respective taxon did not survive to the end of any of the community replicates for the respective evolution temperature. Pairs of evolution temperatures that differ significantly in their intercepts are denoted with X, Y, or Z to denote the pair, and ***p < 0.001, **p < 0.01, and *p < 0.05. Note that the ancestors’ data are only included across all evolution temperatures to facilitate visual comparison to evolved isolates.

As well as investigating the broad-scale effect of the community environment on TPC evolution, we also conducted within-taxon analyses (Figure 2). Here, we used separate GAMMs for each taxon to model the maximum growth rate as a function of treatment group (ancestral, monoculture evolved, community evolved), evolution temperature, and growth temperature to explicitly assess taxon-level variability in TPC evolution.

#### Effects of community context

Treatment group (ancestral, monoculture evolved, or community evolved) was present in the best model for all taxa as either an intercept effect or as a term allowing the smoothing effect on growth temperature to vary between treatment groups—meaning that the shape of the TPC differed between ancestral, monoculture, and community evolved isolates. For three out of five taxa, both of these terms were present in the best model (Table 1). We conducted post-hoc significance testing of the pairwise differences between the intercepts of ancestral, monoculture evolved, and community evolved isolates (see Table 2). For all four taxa with an intercept effect of treatment in the best model (*Pseudomonas* spp., *Serratia* spp., *Aeromonas* spp., and *Herbaspirillum* spp.) the community evolved isolates had a significantly higher growth rate than the ancestor, and for *Pseudomonas* spp. and *Aeromonas* spp. the community evolved isolates also had significantly higher growth rates than the respective monoculture-evolved isolates. For *Serratia* spp. and *Herbaspirillum* spp., monoculture-evolved isolates also had significantly higher growth rates than the ancestor but were not significantly different from their community-evolved counterparts. These findings suggest that for *Pseudomonas* spp. and *Aeromonas* spp. the community environment selects for higher growth than the monoculture environment, yet for *Serratia* spp. and *Herbaspirillum* spp., it does not.

A fixed effect that allows the smoothing term on growth temperature to vary by treatment group was present in the best model for *Serratia* spp., *Aeromonas* spp., *Herbaspirillum* spp., and *Janthinobacterium* spp. This suggests that in addition to contributing to a blanket change in growth rate across the TPC, community context can also alter the shape of the TPC. These community context-mediated changes in TPC shape are variable and taxon-specific. For *Serratia* spp. and *Janthinobacterium* spp., there is a shift toward peak growth occurring at higher temperatures for community-evolved isolates compared to monoculture-evolved isolates. For *Aeromonas* spp., we see the difference in growth rates between monoculture and community-evolved isolates increases around the temperature at which peak growth occurs and decreases at more extreme temperatures. For *Herbaspirillum* spp., the effect is subtle, but community-evolved isolates have a seemingly flatter TPC than monoculture-evolved isolates. We propose the reason that monoculture and community TPCs did not differ in shape in the “All taxa” analysis is related to this variability; when summed, these idiosyncratic and taxon-specific effects pull the aggregate, treatment-level effects in opposite directions, meaning the treatment effects on TPC shape are only revealed by modeling each taxon separately.

The taxon-specific effects of the biotic environment on evolutionary trajectories we observed in our experiments are consistent with previous observations made by Lawrence et al. (2012) in their work investigating resource use evolution (Lawrence et al., 2012). When we consider the complexity of selective pressures imposed by the community background, the diversity in responses from different taxa is unsurprising. In the community treatment group, each taxon will be exposed to a unique set of pairwise interactions with other community members. These interactions will also vary across the time course of the evolution experiment as each community member evolves and relative abundancies shift. Moreover, consideration of pairwise interactions alone is insufficient for predicting evolutionary trajectories in community contexts (Terhorst et al., 2018). Higher-order interactions and indirect effects are also known to be key factors shaping community structure, whereby pairwise interactions between two taxa are altered by the presence of additional taxa (Strauss, 1991). Furthermore, our experiment involved evolution across a thermal gradient, which adds yet another level of complexity because the taxonomic composition, interaction structure, and strength of interactions will vary along the temperature gradient; the community members will also differ in their levels of preadaptation to different temperatures. This multitude of factors means that the complex selective landscape will also depend on the temperature at which the community evolved. The heterogeneity in responses we observe when taxa are evolved within a relatively simple community context highlights the difficulty in predicting evolution in speciose natural microbial communities and the inadequacy of using insights gleaned from monoculture experiments to do so.

#### Effects of evolution temperature

Evolution temperature was observed in the best model for three taxa (*Pseudomonas* spp., *Serratia* spp., and *Herbaspirillum* spp.); however, post-hoc analysis reveals few significant pairwise differences (Figure 2). For *Pseudomonas* spp., the inclusion of evolution temperature appears to be primarily driven by isolates evolved at 23 °C having higher growth than those evolved at 20 °C, 27 °C, and 30 °C (Figure 2A). Similarly, for *Serratia* spp., those that evolved at 23 °C had significantly higher growth rates than those that evolved at 27 °C and 30 °C (Figure 2B). For *Herbaspirillum* spp. there was a significant interaction term between the evolution temperature and treatment group, suggesting that the effect of the treatment group varies between evolution temperatures. However, this effect is subtle, with the greatest effect being the difference between community-evolved isolates and ancestral isolates at 27 °C and 37 °C (Figure 2C). It is notable that the differences in the TPC for *Herbaspirillum* spp. across evolution temperatures and between community evolved, monoculture evolved, and ancestral isolates are less pronounced than for other taxa, yet less variation in the growth rate of this taxon was observed in general. The lower error in the model fit likely allows for the detection of effects with smaller sizes in this case.

The relatively limited effect of evolution temperature on the relationship between growth temperature and growth rate, compared with the varied and pronounced effects of the biotic environment, suggests that evolution temperature is less impactful on the evolution of thermal performance than the community context. This is somewhat surprising given how previous experiments utilizing monocultures have shown marked differences in the TPCs of higher and lower temperature evolved diatoms (O’Donnell et al., 2018), phytoplankton (Padfield et al., 2016), and bacteria (Tian et al., 2022).

**Figure 3.**
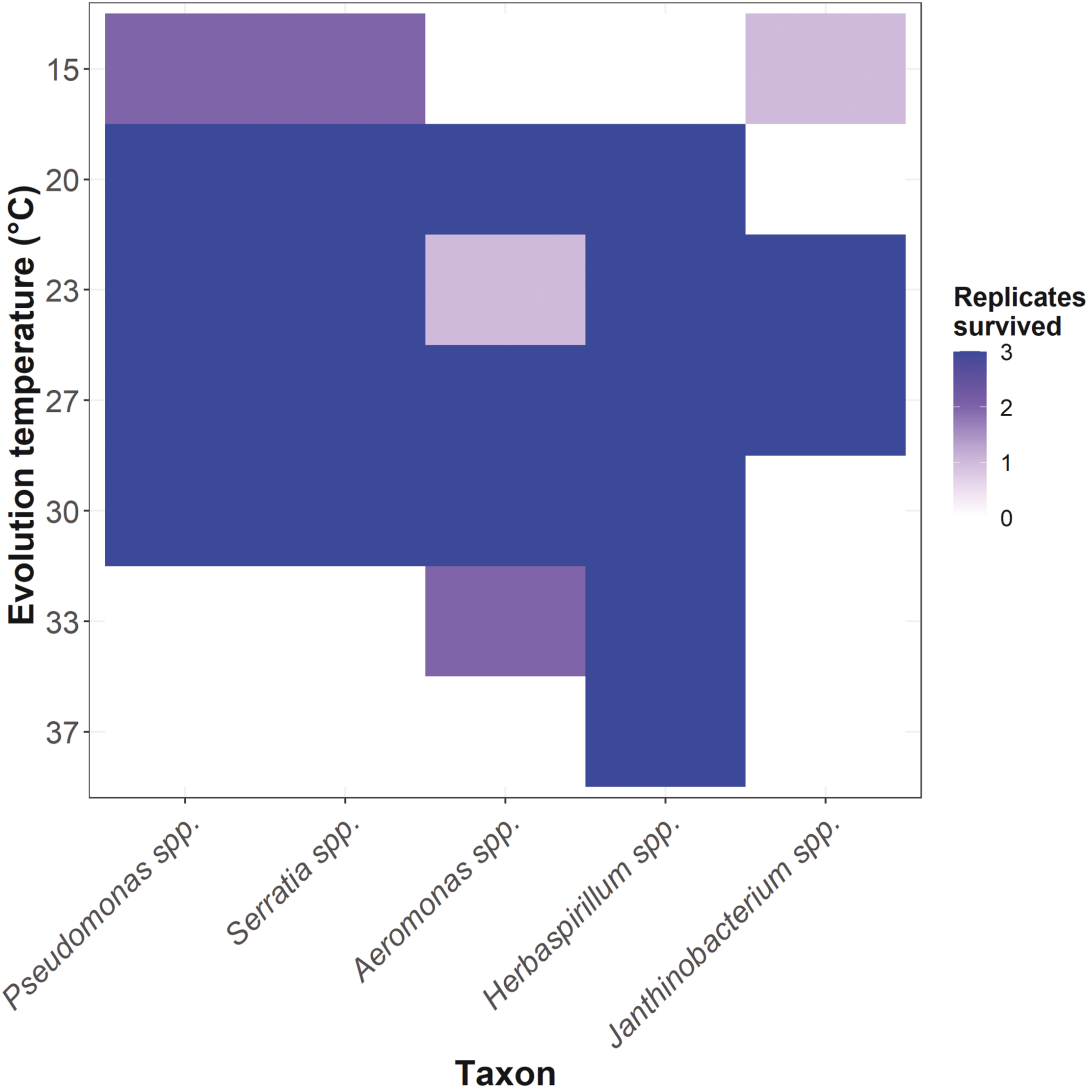
The count of community replicates where each taxon survived to the end of the community evolution experiment at each evolution temperature (°C) out of a total of three.

However, evolution temperature was observed to be related to the local extinction of specific taxa at various temperatures in the communities (Figure 3). The effect of evolution temperature on survival in communities was statistically significant (ΔAICc = 66.356), as was taxon identity (ΔAICc = 13.694). As we observed no extinctions in any monoculture replicates, we hypothesize that these extinctions in the communities are due to competitive exclusion. This temperature-mediated ecological sorting is consistent with previous studies demonstrating that in synthetic laboratory communities, warming alters community structure (Garcia et al., 2022). This is also consistent with field studies showing that experimentally warmed and ambient mesocosms have differing phytoplankton (Yvon-Durocher et al., 2015) and bacterial assemblages (Aydogan et al., 2018). Our results, however, show that temperature-driven extinctions in monoculture are not a prerequisite for local extinctions in a community context. This suggests that in communities, it is the interplay between ecological dynamics imposed by heterospecifics and selection pressure imposed by the thermal environment that regulates whether specific taxa can persist. Furthermore, these findings add significant weight to the hypothesis that temperature change radically alters the ecological context upon which abiotic selection operates by changing the structure of communities.

## Concluding remarks

Here, we provide novel evidence that the community background fundamentally alters the evolution of thermal performance and that this biotic effect has a more pronounced impact on thermal performance phenotypes than the evolution temperature (i.e., the abiotic driver). Furthermore, we show that the effect of the community background is highly idiosyncratic and taxon-specific. Therefore, attempts to predict how microbial communities will respond to global warming will require consideration of the specific community’s composition, how those community members interact, and how the interactions might respond to shifting thermal conditions.

Although, broadly, we see an increase in growth rate across the TPC for community-evolved isolates, by design our study only allows us to observe the evolutionary trajectories of the lineages that persisted to the end of the experiment in the communities. It is likely that these strains persevered precisely because they were able to evolve higher growth rates, increasing their ability to compete with heterospecifics for space and resources. The finding that extinctions only occurred in communities and not in monocultures shows that even in our relatively simple artificial five taxon communities, there is a strong impact of the biotic environment on whether a taxon can persist in the face of shifts in temperature. It is intuitive that in natural communities with very high standing taxonomic richness and continuous exposure to dispersing novel strains, this biotic mediation of the thermal environment’s impact will only be more pronounced.

As microbial systems respond to climate change through the evolution of individual community members and the shifting composition of communities, their contributions to biogeochemical cycling will be altered, potentially considerably. In this study, we provide novel evidence for the extent to which the biotic environment uniquely impacts the evolutionary trajectories and survival of community members undergoing warming. In light of these findings, we argue that the inferences gleaned from monoculture evolution experiments may be insufficient to understand how complex microbial ecosystems will respond to climate change.

## Methods

### Study taxa

Study taxa were derived from biofilm samples collected in May 2016–May 2017 from rock surfaces in several freshwater streams in Hvergerdi Valley, Iceland (64.02, −21.18). These samples were frozen in a 17% glycerol solution after collection and were stored at −20 °C. The freshwater streams from which they originated ranged in temperature from 7 °C to 38 °C due to variation in the levels of geothermal warming at the site (O’Gorman et al., 2014). On return to the laboratory, samples were thawed at 20 °C. The solution they were transported in was then diluted consecutively, and 10 µl of solution was spread onto agar plates and incubated for 10 days at 20 °C. Samples were taken from a random selection of the resulting colonies and were placed into 200 µl of lysogeny broth and incubated for 48 hr. This inoculated lysogeny broth was then centrifuged, and the supernatant was discarded. The pellet of bacterial cells was then placed into a lysogeny broth containing 17% glycerol and was frozen at −80°C.

16S PCR was performed for these samples, and the resulting rRNA was sequenced using Sanger sequencing, and taxonomy was assigned by comparing these sequences with existing databases (see García et al., 2018). The specific methodology follows A master-mix solution was created and consisted of 7.2 μl of DNA-free water, 0.4 μl of 27 forward primer, 0.4 μl of 1492 reverse primer, and 10 μl of Taq polymerase per sample. A template solution was prepared by adding 2 μl of the sample diluted 100 × in DNA-free water to 18 μl of master-mix solution. These samples were then placed in a thermal cycler (Applied Biosystems Veriti Thermal Cycler). The cycling protocol consisted of 1 cycle at 94 °C for 4 min, 35 cycles at 94 °C, 48 °C, and 72°C for 1 min, 30 s, and 2 min, respectively, and finally, 1 cycle at 72 °C for 8 min. The final product of the PCR was cleaned using Exonuclease I and Antarctic Phosphatase. Sanger sequencing was conducted on high-quality samples using the 27F, 1492R primers (Core Genomic Facility, University of Sheffield). Geneious version 6.1.8 (Kearse et al., 2012) was used to trim the sequences, removing the bp from the 5ʹ end and trimming the 3ʹ end to a maximum length of 1,000 bp. Sequences longer than 974 bp were then aligned to the Silva.Bacteria.Fasta database using Mothur version 1.39.5 (Schloss et al., 2009) and the RDP trainset 9 032012 were used as a reference database to assign taxonomy to the isolates. A total of 36 different taxa were identified, and five of them were chosen for use in this study. These five taxa were chosen as they differed in their thermal traits and colony morphologies, the latter requirement being to facilitate visual identification when cultures consisting of more than one taxon were grown on agar. The five taxa chosen for this study and the Genbank accession number were *Pseudomonas* spp. (w_Ic161A, MZ506751), *Serratia* spp. (h_Ic174, MZ506746), *Aeromonas* spp. (n_Ic167, MZ506748), *Herbaspirillum* spp. (j_Ic165, MZ506747), and *Janthinobacterium* spp. (h_Ic161A, MZ506745).

### Evolution of bacteria in monocultures and communities

Bacterial communities comprising all five taxa, as well as monocultures of each taxon, were evolved at temperatures ranging from 15 °C to 42 °C for ~110 generations. We used 110 generations as past research suggests this would be ample time for the communities to reach an equilibrium. In a previous community evolution experiment conducted at 20 °C, it was observed that the majority of communities reached stability after approximately 50 generations (García et al., 2023). Earlier investigations passaging natural communities indicated that around 60 generations were needed for most communities to achieve population equilibria in various instances (Goldford et al., 2018). In the current study, we collected “initial” growth rate data following 2–3 transfers (~10 generations) to allow communities to acclimate to the temperature (mainly to avoid acute stress responses). We then subsequently gathered data at approximately 100 generations later (~110 generations total). The time to reach this number of generations was calculated for the colder evolution temperature groups to ensure all treatment groups reached a minimum of ~100 generations.

The specific methodology follows: An initial stock solution for each taxon was created from a single colony clone using lysogeny broth, which was then incubated overnight at 20 °C. These were then standardized to a common optical density with R2 media, and then a community stock solution was constructed by combining 100 µl of each of the five taxa. 40 µl of stock solution was then used to inoculate 5000 µl of R2 media. Three replicates of these inoculated media were then incubated at each of the following temperatures: 15 °C, 20 °C, 23 °C, 27 °C, 30 °C, 33 °C, 37 °C, and 42 °C. This was then repeated, but instead of inoculation with community stock solution, monoculture stock solution was used, ensuring the same starting biomass of each taxon for each treatment group. Every 48 hr during incubation, 40 µl was removed from each culture and was used to inoculate a fresh 5,000 µl of R2 media to prevent resource limitation from occurring. This was done 18 times, equating to ~110 generations. At the end of the experiment, serial dilutions of the resulting cultures were then grown on agar, and samples of individual taxa were isolated and frozen at −80 °C in 17% glycerol. For the community cultures, individual taxa were identified based on colony morphology.

### Growth assay of evolved isolates

From every evolution experiment, a single clone was isolated. These isolates, as well as the original ancestral samples, were then grown at temperatures ranging from 15 °C to 42 °C. Maximum growth rates [*r*(h^−1^)] were calculated at each temperature. The specific methodology follows:

Every evolved isolate, as well as the original ancestral taxa, were thawed in R2 growth media at 20 °C for 24 hr. These cultures were then diluted with more R2 media until all cultures were at an optical density (OD_600_) of 0.05, measured using a Thermo Scientific Multiskan Sky Microplate Spectrophotometer at a wavelength of 600 nm. Two hundred microliters of each culture was then transferred into 96 well plates. Control “blank” wells were filled with only R2 medium. The plate was then incubated at 15 °C until carrying capacity was reached (~54 hr), and OD_600_ measurements were taken every ~4 hr. This process was repeated for all isolates at incubation temperatures of 20 °C, 23 °C, 27 °C, 30 °C, 33 °C, 37 °C, and 42 °C. Due to handling time, there was some variation in measurement intervals, but in all analyses, exact intervals calculated from timestamps were used. The mean OD_600_ value for blank wells in a plate was subtracted from all OD_600_ measurements.

### Fitting growth curves

All modeling of growth curves was conducted in R version 4.0.2 (R Core Team, 2021) using the nlsLoop package (Padfield, 2016/2020). The maximum growth rates [*r* (h^−1^)], hereafter simply *r*, or maximum growth rate) for each incubated culture were calculated by fitting the logistic growth equation to the OD_600_ measurements, using nonlinear least squares regression.


$$\begin{array}{l} N_{t}=\frac{K}{1+Ae^{-rt}};A=\frac{K-N_{0}}{N_{0}}\; \end{array}$$ (1)

In Equation 1, *N*_*t*_ is the biomass at time, *t*, *K* is the carrying capacity, *N*_0_ is the starting biomass, and *r* is the exponential population growth rate [*r*(h^−1^)]. For some cultures, after reaching carrying capacity, there would be a slow decline in cell density. As the above model cannot estimate this decline, these data points demonstrating a postasymptote decline were removed.

### Statistical analysis

All statistical analyses were conducted in R version 4.0.2 (R Core Team, 2021), and all plots were created using *ggplot2*, and other *tidyverse* (Wickham et al., 2019) packages were used for data handling. For all analyses, monoculture-evolved isolates were only included if their respective isolates had survived to the end of the community evolution experiment. For example, at an evolution temperature of 33 °C, only *Aeromonas* spp. and *Herbaspirillum* spp. survived to the end of the evolution experiment; therefore, only *Aeromonas* spp. and *Herbaspirillum* spp. isolates that evolved at 33 °C in monoculture were included in the analysis, and *Pseudomonas* spp., *Serratia* spp., and *Janthinobacterium* spp. isolates that evolved at 33 °C in monoculture were excluded from the analysis. This prevented a survivorship bias from confounding the results.

For the within-taxon analyses, separate generalized additive mixed-effect models (GAMMs) were fit for each taxon, using the function *uGamm* from the R package *MuMIn* (Bartoń, 2022). The initial full model included maximum growth rate as the response variable and the following fixed effects and smoothing terms: evolution temperature, treatment (monoculture, ancestral, or community evolved), an interaction between evolution temperature and treatment, and a smoothing term on growth temperature, which was allowed to vary by treatment. A single random effect encompassing taxon and biological replicate (at the level of each evolved lineage) was included in all models. All possible sub-models were created and compared using their sample corrected Akaike information criterion (AICc) using the function *AICc* from the package *MuMIn*, although models without a smoothing term on growth temperature were not considered. The threshold for determining a significant difference between models was when ΔAICc was >2. Where there were one or more models falling within 2 ΔAICc of the lowest AICc model, the more minimal model was selected as the best model. The R package *emmeans* (Lenth, 2017/2023) was used to conduct post hoc pairwise comparisons for model estimates across both evolution temperatures and treatment groups.

For the “all taxa combined” analysis, model creation and selection were conducted in the same way as the within-taxon analyses. Fixed and random effects were the same as in the within-taxon analyses. However, due to issues with rank deficiency when trying to incorporate ancestral data into this model, a separate model for the effect of growth temperature on the growth rate for the ancestor was constructed. This negates pairwise significance testing of differences between ancestral and evolved lineages but does allow visual comparison of TPCs by superimposing the ancestral model predictions onto a figure displaying the predictions for the monoculture and community evolved isolates (Figure 1).

For the community survival analysis, binomial generalized linear models were fit using the base R *glm* function, and the fixed effects "taxon identity" and "evolution temperature". Fixed effects were determined to be significant if the ΔAICc of their removal was >2.

## Data Availability

All data and code used to replicate the figures, analyses, and findings in this paper can be found at https://doi.org/10.5061/dryad.vq83bk41b.
